# Neuro-computational account of how mood fluctuations arise and affect decision making

**DOI:** 10.1038/s41467-018-03774-z

**Published:** 2018-04-26

**Authors:** Fabien Vinckier, Lionel Rigoux, Delphine Oudiette, Mathias Pessiglione

**Affiliations:** 1 Motivation, Brain and Behavior lab, Centre de NeuroImagerie de Recherche, Institut du Cerveau et de la Moelle épinière, Hôpital de la Pitié-Salpêtrière, 47 Boulevard de l’Hôpital, Paris, 75013 France; 2 Inserm U1127, CNRS U7225, Université Pierre et Marie Curie (UPMC-Paris 6), Hôpital de la Pitié-Salpêtrière, 47 Boulevard de l’Hôpital, Paris, 75013 France; 3 Department of Psychiatry, Service Hospitalo-Universitaire, Centre Hospitalier Sainte-Anne, 1 rue Cabanis, Paris, 75014 France; 4 Sorbonne Paris Cité, Université Paris Descartes (Paris-V), 12 Rue de l’École de Médecine, Paris, 75006 France; 5 Laboratoire de Physiopathologie des Maladies Psychiatriques, Inserm UMR S894, Centre de Psychiatrie et Neurosciences, 1 rue d’Alésia, Paris, 75014 France; 6 Translational Neurocircuitry Group, Max Planck Institute for Metabolism Research, Cologne, 50931 Germany; 7 Translational Neuromodeling Unit, Institute for Biomedical Engineering, University of Zurich and ETH Zurich, Wilfriedstrasse 6, Zurich, 8032 Switzerland; 8 Service des Pathologies du Sommeil, Hôpital de la Pitié-Salpêtrière, 47 Boulevard de l’Hôpital, Paris, 75013 France

## Abstract

The influence of mood on choices is a well-established but poorly understood phenomenon. Here, we suggest a three-fold neuro-computational account: (1) the integration of positive and negative events over time induce mood fluctuations, (2) which are underpinned by variations in the baseline activities of critical brain valuation regions, (3) which in turn modulate the relative weights assigned to key dimensions of choice options. We validate this model in healthy participants, using feedback in a quiz task to induce mood fluctuations, and a choice task (accepting vs. declining a motor challenge) to reveal their effects. Using fMRI, we demonstrate the pivotal role of the ventromedial prefrontal cortex and anterior insula, in which baseline activities respectively increase and decrease with theoretical mood level and respectively enhance the weighting of potential gains and losses during decision making. The same mechanisms might explain how decisions are biased in mood disorders at longer timescales.

## Abstract (web summary)

Fluctuations in mood are known to affect our decisions. Here the authors propose and validate a model of how mood fluctuations arise through a slow integration of positive and negative feedback and report the resulting key changes in brain activity that modulate our decision making.

## Introduction

People gamble more on a surprisingly sunny day or after the unexpected victory of their favorite sport team^1–4^. Such facts are classically considered as evidence for the influence of mood on judgment and decision-making, which is also well supported by clinical observations. Depressed patients tend to neglect positive aspects, whereas manic patients tend to ignore the potential negative consequences of their actions^5–7^. Mood-congruent judgments have also been repeatedly observed in the lab^8–10^, with the consistent finding that mood enhancement favors risk taking^11–13^. Yet the mechanisms underlying this pervasive phenomenon are poorly understood. Two key insights have been suggested recently: first a computational account of how mood fluctuations could arise from the feedback that a person receives^14–16^, second the demonstration that pre-choice activity in specific brain regions exerts a bias on value-based judgment and choice^17,18^. The aim of the present study is to put these pieces together, and to extend current understanding by explaining how mood distorts the valuation process underlying choice. In short, our objective was to provide a comprehensive neuro-computational model of how mood fluctuations arise and influence choice under risk.

The first step was to account for how history of feedback translates into fluctuations in mood level. For this part, we took inspiration from recent suggestions that mood level reflects a weighted integration of expectations and outcomes, with an exponential decay over time^14^. Such simple models provide a good fit of mood (or momentary happiness) ratings provided by participants playing with slot machines. Later models have included a reciprocal influence of mood on the perception of feedback^15^, such that good mood induces a “rosy outlook”, taking events as more positive than they objectively are. The two-way interaction between mood and feedback was shown to bias the learning of payoffs associated with slot machines, particularly in subjects with hypomanic personality. Importantly, using computational modeling enables tracking the neural correlates of mood level without asking subjects to rate their mood, which could by itself interfere with the choices.

The second step was to identify the brain regions expressing mood level in their baseline activity, during the rest period between last feedback and next choice. To our knowledge, previous studies did not exactly address that question, but rather looked for neural responses to stimuli triggering emotional reactions^19–22^ that are short-lived compared to mood states. Indeed, mood should fluctuate at a longer timescale, and should be less tightly linked to a single event. However, because mood level varies with positive and negative feedback, candidate regions could be inferred from the literature on reward vs. punishment (or appetitive vs. aversive) processing. On the one hand, the reward circuit mainly includes the limbic frontostriatal pathway, linking the ventromedial prefrontal cortex (vmPFC) to the ventral striatum (vS), under the influence of mesocorticolimbic dopaminergic projections^23,24^. This pathway has also been implicated in the so-called brain valuation system, whose activity positively correlates with the value of various items such as money, food, music, painting, faces etc.^25–28^ On the other hand, the pain matrix mainly includes the anterior insula (aIns) and anterior cingulate cortex (ACC). It is known to activate with aversive stimuli, from pain to social rejection^29–32^. Thus, a priori candidates for reflecting mood level were vmPFC and vS (with positive correlation) and aIns and ACC (with negative correlation).

The third step was to explain how mood-related baseline activity could influence choice under risk. Previous studies have shown that brain activity preceding stimulus presentation could bias valuation and choice. For instance, higher firing rate in monkey VMPFC was shown to predict more lipping (an appetitive Pavlovian reflex) in response to reward-associated cues, and similarly higher functional magnetic resonance imaging (fMRI) activity in human VMPFC was predictive of paintings being later judged as more pleasant^18^. In another monkey study, pre-stimulus activity of single neurons in the orbitofrontal cortex was predictive of an additional bias on choices between reward-associated cues on top of their subjective value^17^. Also, pre-stimulus multivariate activity in vS and medial frontal cortex was predictive of the propensity to take risk in a lottery task^33^, and anticipatory neural activity in vS vs. aIns was predictive of risk-seeking vs. risk-averse suboptimal choice, reciprocally^34^. However, these effects of pre-stimulus activity were independent of stimulus content and could not explain how mood-related baseline activity influences the integration of the different features of choice options. To specify this influence, we first adapted a normative choice model from expected utility theory to our choice task and then investigated which parameters of this model may be affected by mood-related baseline activity.

Our results support a neuro-computational account in which feedback-induced mood fluctuations are underpinned by baseline activity in relevant valuation networks (notably vmPFC and aIns), which in turn affects the relative weights assigned to the different dimensions of choice options (notably gain and loss prospects).

## Results

### General approach

**Fig. 1:**
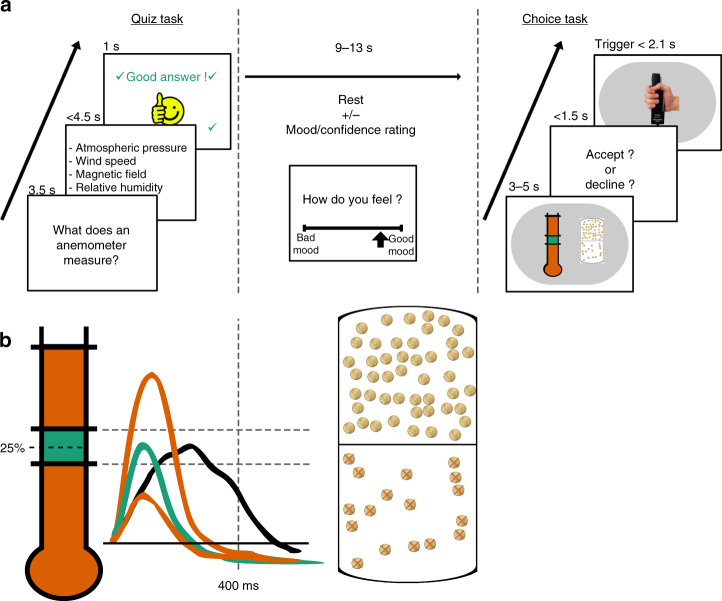
Behavioral tasks. **a** Trial structure. Each trial included two unrelated tasks. In the quiz task (left), participants had to select one of four possible answers to a general knowledge question. In the choice task (right), participants had to decide between accepting or declining a motor challenge. The two tasks were separated by a rest period. In a subset of participants, subjective ratings of mood (or confidence) were inserted into the rest period. **b** Motor challenge. The challenge consisted in squeezing a handgrip such that the force peak would fall within a window around 25% of their maximal force. The green line represents a successful trial. Participants could fail because they squeezed too much (upper red line), not enough (lower red line) or for too long (more than 400 ms, black line). The challenge varied across trials along three dimensions that were indicated at the time of choice: difficulty, represented by the size of the target window (in green on a red thermometer), expected gain in case of success, represented by a bunch of regular 10-cent coins (in the upper part) and expected loss in case of failure, represented by a bunch of 10-cent crossed coins (in the bottom part). If subjects declined the challenge, they still had to squeeze the handgrip and try to hit the target window, but the stakes were minimized (expected gain and loss were fixated at 50 cents)

In the present study, participants performed two unrelated but interleaved tasks, separated by a few seconds rest period. The first was a quiz task used as a mood induction procedure^35,36^, the second was a choice task used to unravel the effects of mood induction on decision-making (Fig. 1). In the quiz task, participants had to answer general knowledge questions and received feedback (correct or incorrect response). Unbeknownst to them, quiz difficulty and feedback were biased so as to create episodes of high and low correct response rate. In the choice task, participants had to decide whether to accept or decline a motor precision challenge, consisting in squeezing a handgrip so as to hit a force target (around 25% of maximal force). Difficulty level (target size), gain prospect (in case of success), and loss prospect (in case of failure) were varied on a trial-by-trial basis. In order to avoid learning effects, and additional effects on mood, no feedback was provided regarding motor precision. Participants were explicitly informed that the two tasks were independent, such that responses in the quiz had no influence onto difficulty level or monetary prospects.

Three independent datasets (*n* = 15, 23, and 23 participants) were acquired, with minor modifications of the design (see Methods section for details). The first dataset was acquired during a pilot study conducted in order to adjust the choice model. The second dataset was acquired using a variant of the task where ratings of mood (how well subjects felt) were inserted during the rest period between quiz and choice tasks, in order to adjust the mood model. We also included confidence ratings (about success in the next motor challenge), to confront the specificity of the mood model. A theoretical mood level (TML) could then be inferred from the history of tasks events, using the mood model. The third dataset was acquired during an fMRI study, in which we identified the neural correlates of TML and their effects on the parameters of the choice model. Thus, the origins and consequences of mood fluctuations were explored without subjects having to report their mood level, which could have artificially determined their choice behavior.

**Fig. 2:**
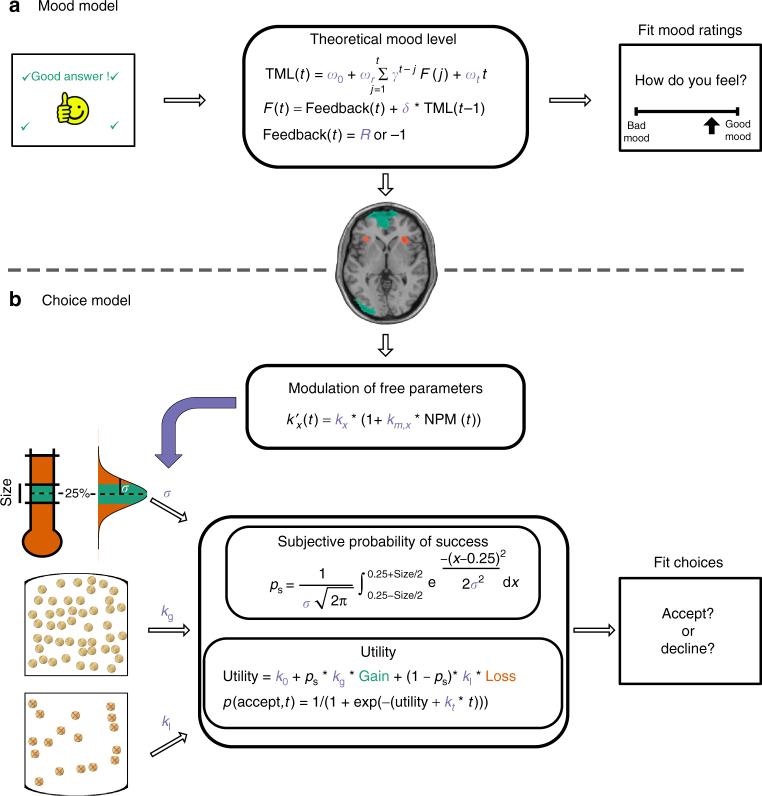
Computational modeling. The figure illustrates the best models that were selected using group-level random-effect analyses. **a** Best mood model. Theoretical mood level (TML) was computed as an integration of quiz feedback, with an exponential decay. This feedback was subjective, as its perception was in return biased by TML, and different for positive and negative feedback. TML was then regressed against fMRI data to identify the neural correlates of mood during the rest period between the quiz and choice tasks. **b** Best choice model. Baseline activity (at the time of prospect display) in mood-related regions (termed Neural Proxy for Mood, NPM) modulated the free parameters of the choice model. A first parameter was the variance of the subjective distribution of forces produced (parameterized by *σ*, the width of the Gaussian), which served to calculate the subjective probability of success *p*_s_, as the integral of probability distribution within the target window. This probability *p*_s_ was integrated in the expected utility of the prospect, which had two other free parameters—the weights of potential gain and loss, *k*_g_ and *k*_l_. Acceptance probability was calculated as a sigmoid function (softmax) of expected utility, with a last parameter *k*_*t*_ that accounted for a linear drift with time

The aim of data analysis was to provide a neuro-computational description of how mood fluctuations arise and affect choices. Our general strategy (illustrated in Fig. 2) was (1) to build separate models of choice and mood level that provide the best trade-off between accuracy and complexity, (2) to identify the neural markers of TML predicted by the mood model, and (3) to specify how these neural markers would modulate the parameters of the choice model.

### Computational model of choice

**Fig. 3:**
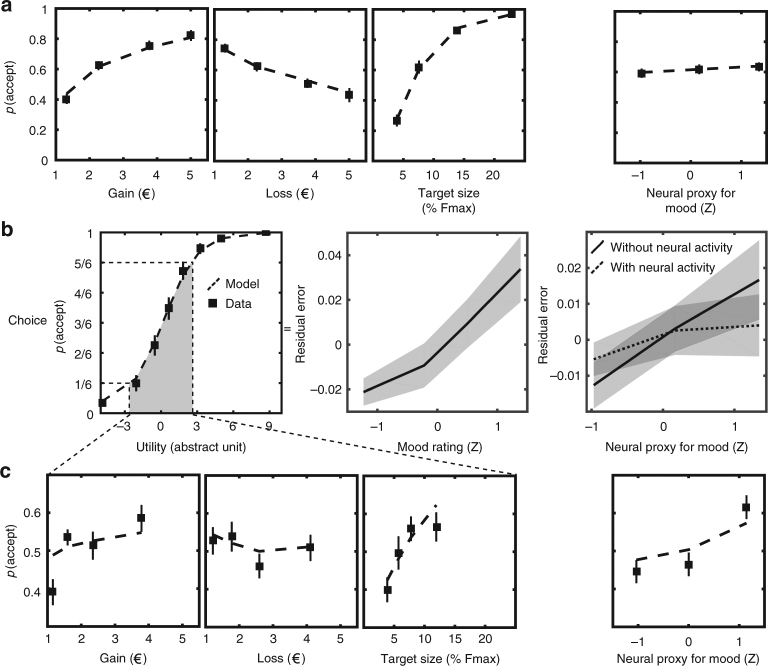
Choice behavior. The different panels show the effects of experimental factors and neural activity on the probability to accept the motor challenge (in experiment 3, unless indicated otherwise). **a** Acceptance probability as a function of the prospect dimensions (gain, loss, target size) and the neural proxy for mood (NPM). Squares are binned data points. Dashed line represents acceptance probability as computed by the best choice model. **b** Left panel: acceptance probability as a function of expected utility. Dashed line represents acceptance probability as computed in the best choice model (without modulation of free parameters by neural activity). Middle panel (experiment 2): Residual error of choice (actual choice – acceptance probability) as a function of mood rating. Right panel: Residual error of choice (actual choice – acceptance probability) as a function of NPM for the best choice model with (dashed line) and without (solid line) modulations of *k*_g_ by vmPFC baseline and *k*_l_ by aIns baseline. Note that these modulations captured most of the influence of NPM on the variance in choices that was not explained by task factors. **c** Acceptance probability as a function of prospect dimensions and NPM, but restricted to trials for which acceptance probability (as computed by the best choice model without modulation of free parameters by neural activity) was between 1/6 and 5/6. Here the effect of baseline activity in mood-related regions was of a similar size as the effects of task factors. Error bars represent inter-subject s.e.m.

Choice data were acquired in all three experiments. Before modeling, we checked that the three dimensions of prospects (target size, gain, and loss) were significantly integrated into choices (all *p*-values < 0.001, two-tailed *t*-tests, Fig. 3a). The aim of computational modeling here was to provide the best possible account of how the three dimensions were combined to generate choices.

The best model according to Bayesian model selection (see Methods section) was the same in all three datasets (exceedance probability xp = 0.76, 0.73 and 0.95 for experiments 1, 2 and 3 respectively; xp = 0.99 when pooling the three datasets). This model (detailed in Fig. 2, fit illustrated in Fig. 3) is based on expected utility theory, with Utility(accept) calculated through multiplication of potential gain and loss by probability of success vs. failure (inferred from target size), the gain and loss terms being weighted by distinct parameters (*k*_g_ and *k*_l_) but without any curvature (no power parameter). The absence of curvature might relate to the fact that gains and losses were maintained within a limited range (1–5€). Utility(accept) is then compared to a constant Utility(decline) in a softmax function to calculate probability of acceptance. This indicates that subjects were only estimating Utility(accept) from the new prospect proposed every trial, and were neglecting the variations of Utility(decline) related to the changes in target size.

We used the choice model to regress out the prospect factors (gain, loss, and target size) and check that mood was indeed predictive of the willingness to accept the motor challenge. The residual error of choice model fit was thus regressed against mood rating. This association was significantly positive at the group level (*t*(22) = 2.3, *p* = 0.028, Fig. 3b), indicating that mood rating explain choice above and beyond the factors manipulated in the choice task.

### Computational model of mood

**Fig. 4:**
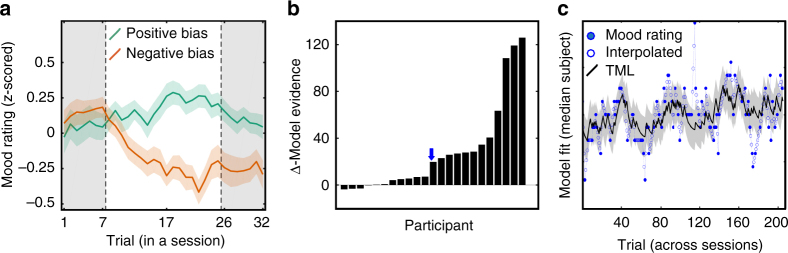
Mood fluctuations. **a** Variations in mood rating during sessions with positive (green) vs. negative (red) bias, across trials. Bias here means more or less difficult questions, and more or less correct feedback. Note that the seven first and seven last questions of a session (grey windows) were not biased (difficulty was medium). Lines represent means; error bars represent inter-subject s.e.m. The other panels show how well the computational model captured fluctuations in mood level. **b** Individual differences in model evidence (variational Bayesian approximation to marginal likelihood) between the best mood model and the control model (in which only time was taken into account). Participants were ranked from left to right in ascending order of model evidence. Blue arrow indicates the subject with median model evidence, plotted in the next panel. **c** Individual example of mood fluctuations across trials. Blue circles are mood ratings (measured or interpolated) and black line is theoretical mood level (TML). See Supplementary Fig. 1 for other individual examples

Before modeling, we verified that our mood induction procedure was efficient. Indeed, mood was higher during task episodes biased toward more frequent correct feedback, relative to less frequent correct feedback (*t*(22) = 3.7; *p* = 0.001, Fig. 4a).

The aim of computational modeling here was to provide the best possible account of how the variables manipulated in the quiz and choice tasks affected the mood and/or confidence ratings recorded in experiment 2. Following on previous work^14^, we expected mood to be influenced not only by outcomes but also by expected value (EV) and reward prediction error (RPE) from both tasks, an influence that would decay exponentially with time.

However, the best model (xp = 0.97, Fig. 4b, c and Supplementary Fig. 1) according to Bayesian model selection (see Methods section) was a simplified one in which only the quiz task (and not choice task) was influential, through an influence of feedback only, this influence being asymmetric for positive and negative feedback. Interestingly, as in previous studies, this best model formalizes a reciprocal influence between mood and feedback (see Equations in Fig. 2): feedback weight *ω*_f_ was significantly positive across participants (*t*(22) = 4.3, *p* < 0.001), meaning that positive feedback induced better mood, and so was mood weight *δ* (*t*(22) = 2.8, *p* = 0.01), meaning that better mood led to perceiving feedback as better than it was. Parameter *ω*_*t*_ was significantly negative (*t*(22) = −3.0, *p* = 0.006), indicating that mood level globally decreased with time on task.

In contrast, the best model identified for confidence ratings was the null, in which no task-related variable had any influence (xp = 0.80). This suggests that the quiz task did impact mood and not what subjects reported as their confidence in the motor precision task. We therefore focus on mood fluctuations (as approximated by the TML generated by the best model) in the following, and looked for neural correlates in the fMRI data, which were recorded in the absence of mood ratings (experiment 3).

### Brain activity underpinning mood fluctuations

The best mood model, with parameters corresponding to posterior means averaged across participants in experiment 2, was applied to the individual history of feedback in order to generate trial-by-trial TML for each participant in experiment 3 (where there was no mood rating). To assess whether mood level could be reflected in post-feedback activity of critical brain regions, we included TML as a parametric modulator of the rest period between the quiz and choice tasks (modeled as a boxcar), in GLM used to analyze fMRI data. Note that the rest period corresponds to the time window where mood level was probed in experiment 2. The general linear model (GLM) also included quiz question, answer and feedback onsets, as well as prospect onsets with its expected utility (generated by the best choice model) as a parametric modulator.

**Fig. 5:**
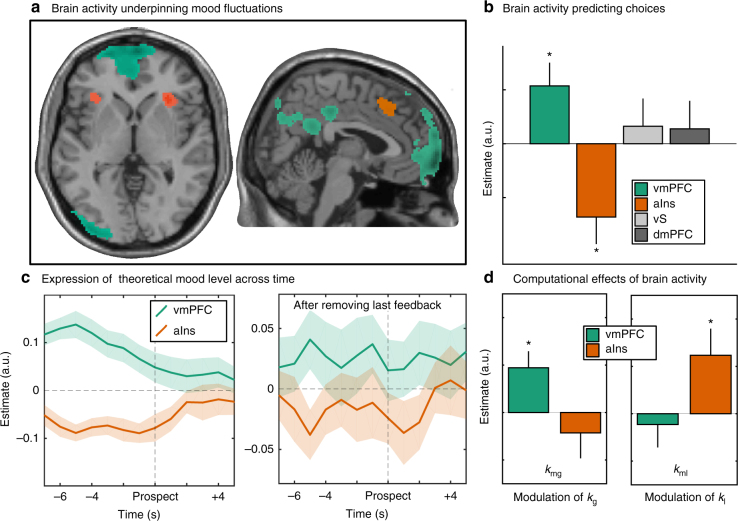
Neural activity. The different panels show how baseline activity in key valuation regions reflects mood (top line) and affects computational parameters of the choice model (bottom line). **a** Statistical parametric map of regions reflecting TML during rest (positive correlation in green, negative correlation in red, cluster generating threshold *p* < 0.001, cluster size arbitrarily defined for display purpose). Four ROI (vmPFC, aIns, vS, and dmPFC) were defined using spheres of 8-mm diameter centered on coordinates from the literature (coordinates from ref. ^42^). **b** Impact of ROI activity on choice. Baseline activity was extracted for each subject and trial from our 4 a priori ROI, and orthogonalized with respect to whole-brain mean activity. The 4 trial-by-trial series were then entered in a linear regression model meant to explain the residual error of the choice model fit. Only vmPFC and aIns were significant predictors, with opposite directions. **c** Contribution of vmPFC and aIns to TML. Baseline activity was extracted at each time point and entered in a linear regression model meant to predict TML. Both vmPFC and aIns were significant predictors of TML, with opposite directions, even when all variables were orthogonalized with respect to the last feedback. **d** Impact of ROI activity on choice model parameters. Significant modulation was only found with vmPFC for *k*_g_ (weight on potential gain) and with aIns for *k*_l_ (weight on potential loss). Bars represent means of parameters estimates; error bars represent inter-subject s.e.m.

At the whole-brain level (cluster-generating threshold *p* < 0.001, cluster-level threshold *p* < 0.05 family-wise error corrected), we found positive correlation with TML in a network encompassing visual cortex, left inferior parietal lobule, posterior cingulate cortex and vmPFC, and negative correlation in the dorsomedial prefrontal cortex (dmPFC) and left frontal inferior gyrus (Fig. 5a, Supplementary Table 1, see also Supplementary Fig. 2 for response to feedback). Two of these regions corresponded to our a priori regions of interest (ROI): vmPFC and dmPFC (which overlaps with a region that has been labeled as dorsal ACC in other studies). In addition, when looking into ROI defined from the literature, we observed a significant negative correlation with TML in bilateral aIns (both *p* < 0.01), and a marginally significant positive correlation with TML in bilateral vS (both *p* < 0.06). As the linear interpolation applied to mood ratings could affect model parameters, we checked that similar neural correlates are obtained when regressing TML obtained with a mood model estimated on actual ratings only (without interpolated ratings).

### Computational impact of mood-related brain activity

Before modeling, we identified which of our a priori ROI had a significant impact on choice. We extracted baseline activity (at the time of prospect onset) on a trial-by-trial basis in each ROI and included these four regressors in a unique model meant to explain the residual error of the best choice model fit. Activity was averaged across left and right hemispheres for bilateral ROI (aIns and vS), and orthogonalized with respect to whole-brain mean activity for all four ROI. This orthogonalization was performed to ensure that any effect could not be explained by unspecific fluctuations in the blood-oxygen-level dependent (BOLD) signal. At the group level, we found a significant association for the vmPFC (*t*(22) = 2.5, *p* = 0.021) and aIns (*t*(22) = −2.7, *p* = 0.012), but not for vS and dmPFC (both *p* > 0.1, Fig. 5b). This means that not only baseline activity in vmPFC and aIns had opposite influence on upcoming choice, but also these influences were not fully redundant. Finally, we regressed polynomial expansions of TML against vmPFC and aIns activity, to examine the possibility of non-linear links. We found no significant association beyond the first-order (linear) regressor (Supplementary Fig. 3). We also checked that the regression slopes estimated above and below median TML were not different (both *p* > 0.1), as would be predicted if the vmPFC was only reflective positive mood, and the aIns negative mood level.

To assess whether these two regions would also represent separate components of mood, we entered them in a same regression model meant to explain TML. In order to obtain the time course of mood expression in the two ROI, we performed this regression for each time point around prospect display, using FIR models to extract time-series of brain activity. In order to rule out effects of no interest (e.g., time), TML was orthogonalized with respect to trial number, as well as the two neural regressors, which were also orthogonalized to mean brain activity. Regression coefficients obtained for the eight scans preceding prospect onset were then entered in a mixed-effect model with intercept as fixed effect and subject-wise random effects of both intercept and time point. The fixed intercept regressor was a significant predictor of TML for both vmPFC and aIns activity, with opposite signs (βvmPFC = 0.098; aIns = −0.083, both *p* < .001; Fig. 5c, and Supplementary Fig. 3). Moreover, in order to ensure that TML was expressed above and beyond the variance induced by the last feedback, we performed the same analysis after orthogonalizing TML and the two regressors with respect to last feedback (−1 or +1 for TML, feedback-evoked response for vmPFC and aIns activity). Both regions were still independent predictors of TML (βvmPFC = 0.043; aIns = −0.032, both *p* < 0.01; Fig. 5c).

Thus, fMRI data analyses showed that vmPFC and aIns pre-choice activity reflect separate components of mood and have independent effects on subsequent choice. The next step was to specify the computational mechanism by which pre-choice activity could modulate decision making. Previous analyses offered a way to translate brain activity into mood level at the time of prospect display, using the coefficients obtained when regressing TML against vmPFC and aIns activity. We applied this linear model to baseline activity in order to generate a neural proxy for mood (NPM), as follows:

$$\mathrm{NPM} = {{\beta} \mathrm{vmPFC}} \ast {\mathrm{baseline}}({\mathrm{vmPFC}}) + {\mathrm{\beta aIns}} \ast {\mathrm{baseline}}({\mathrm{aIns}})$$

As a sanity check, we verified that NPM was still predictive of choices (*t*(22) = 2.9, *p* = 0.009, Fig. 3). When all choice data were included in the regression, the effect of mood looked quite small (~4% between lowest and highest mood, Fig. 3a) compared to the other factors. However, when restricting the data set to trials in which choices were not too strongly determined by task factors (i.e., trials for which acceptance probability, as computed by the best choice model, was between 1/6 and 5/6), the effect of mood effect was much higher (~15%) and comparable to the other dimensions (Fig. 3c).

In order to specify the mechanism by which mood could interfere with decision-making, we allowed NPM to modulate the free parameters of the best choice model, focusing on how task factors (gain, loss and target size) were integrated (modulation of *k*_g_, *k*_l_, and *σ*). Modulated free parameters were computed as follows:

$$k_{\mathrm{g}}' (t) = k_{\mathrm{g}} \ast (1 + k_{\mathrm{mg}} \ast {\mathrm{NPM}}(t))$$

where *k*_mg_ (respectively *k*_ml_ and *k*_mσ_) is the weight that NPM had on the gain prospect parameter (respectively on loss prospect parameter and subjective variability of force production).

We also allowed NPM to bias expected utility in an additive way, independent from task factors:

$$U_{\mathrm{accept}}' (t) = U_{\mathrm{accept}}(t) + k_{\mathrm{m0}} \ast {\mathrm{NPM}}(t)$$

We started with a Bayesian comparison between the 16 resulting models (each of the four possible modulations—*k*_mg_, *k*_ml_, *k*_mσ,_ and *k*_m0_—could be included or not). No best model emerged but family-wise analyses revealed that modulation of *k*_g_ and *k*_l_ were both plausible (xp = 0.65 and xp = 1), whereas modulation of *k*_mσ_ and additive modulation were both implausible (both xp = 0). Moreover, *t*-tests on posterior means across participants indicated that modulation of *k*_l_ (i.e., *t*-test on *k*_ml_) was significantly negative (*t*(22) = −2.5, *p* = 0.02), meaning that loss weight decreased when NPM was higher. The *t*-test on *k*_mg_ was numerically positive but not significant, yet this was likely due to an outlier subject (−3.3 SD to the mean), since a non-parametric test (Wilcoxon) was significant (*p* = 0.048). This would suggest that not only participants tend to neglect potential losses when their mood is higher, but they tend to overweight potential gains. We next intended to decompose these two effects of NPM and assess whether they could be assigned to our two ROI.

For each ROI, we compared choice models in which baseline activity could modulate *k*_g_, *k*_l_ or both. For vmPFC the best model was a specific modulation of *k*_g_ (xp = 0.96), whereas for aIns the best model was a specific modulation of *k*_l_ (xp = 1). Furthermore, we performed *t*-tests on posterior parameters of the model that included modulation of both *k*_g_ and *k*_l_. We found that *k*_g_ was significantly enhanced by vmPFC baseline (*t*(22) = 2.7, *p* = 0.01), whereas *k*_l_ was significantly enhanced by aIns baseline (*t*(22) = 2.2, *p* = 0.04). The other possible links (*k*_l_ with vmPFC and *k*_g_ with aIns) were negative but failed to reach significance (Fig. 5d).

Finally, we verified that the two critical effects of baseline activity we identified (modulation of *k*_g_ by vmPFC activity and modulation of *k*_l_ by aIns activity) mediated most of NPM effect on choices. When using the best choice model without brain activity, regression of residual error (part of choice unexplained by task factors) with NPM was highly significant (*t*(22) = 2.9, *p* = 0.009), as already described. When including both vmPFC effect on *k*_g_ and aIns effect on *k*_l_ in the choice model, the regression with NPM was only marginally significant (*p* = 0.06), meaning that residual error was roughly flat (Fig. 3b). Moreover, a direct comparison of slopes was highly significant (*t*(22) = 3.3, *p* = 0.004), showing that vmPFC and aIns baseline activities mediated most of NPM effect on choices.

## Discussion

In this paper, we used a computational approach to demonstrate that mood fluctuations induced by irrelevant positive and negative feedback were expressed in the baseline activity of two critical regions, vmPFC and aIns, which in turn biased how gain and loss prospects were weighted when making a decision. More specifically, we found that at choice onset, high vmPFC baseline activity promoted risk taking through overweighting of potential gains, while high aIns baseline activity tempered risk taking by overweighting potential losses. In the following, we discuss successively how mood fluctuations arise from feedback, how they are represented in baseline brain activity, and how baseline brain activity impacts the weighting of prospect dimensions.

Mood was manipulated here through the feedback that participants received in a quiz task. Feedback is arguably a key factor of mood fluctuations but it could be seen as impacting confidence rather than mood, which is also susceptible to external factors (independent from the person’s behavior), such as having nice vs. bad weather, or watching a happy vs. sad movie. Yet we found that the history of feedback affected what participants reported as their mood level, and not their confidence level. The way feedback was integrated into our winning computational model is close to the suggestion of Rutledge and colleagues^14^, with a leaky accumulation across trials (sum weighted by exponential decay). Critically, the influence of feedback on mood was reciprocated, feedback being more positively perceived when mood was better. This corresponds to the common intuition, captured by Eldar and Niv’s model^15^, that happy people have a rosy outlook. The main difference was that in their model, mood followed prediction errors (outcome minus expectation), rather than just feedback. This discrepancy is likely to result from participants having no precise expectation in our quiz task (contrary to their bandit tasks), or from us having no precise estimate of participants’ expectations. The best proxy that we could find for expectation was mean performance over subjects (correct response rate), which was probably too much of an approximation for prediction-error models to win the comparison. Another difference was that the impact of positive and negative feedback was asymmetrical in our model. This is reminiscent of the asymmetrical effect of dopaminergic medication on reward and punishment outcomes^37–39^, which may suggest a role for dopamine in mediating the effects of feedback on mood.

Positive and negative feedback had quite a long-lasting effect on mood ratings: discount factor was 0.77 on average, meaning that a feedback received five trials in the past had an impact corresponding to 35% of the most recent feedback. This was also the case at the neural level: vmPFC and aIns baseline activities reflected TML above and beyond the last feedback, and therefore could not be interpreted as evoked responses. Yet we must acknowledge that as mood was only sampled every three trials, we had to interpolate ratings in order to get sufficient statistical power for model comparison. This linear interpolation may have influenced the dynamics of mood fluctuations captured by our model, so our estimate of the discount factor must be taken with caution. In any case, the timescale of mood fluctuations in our study was around the minute, which is rather short compared to spontaneous affective fluctuations, whose timescales range from hours to days^40,41^. This may question the validity of our computational model for capturing ecological mood fluctuations. An answer to that critique is the demonstration that a similar model could apply to real-world incidental events, such as sport results or weather forecasts, and generated a proxy for mood level that improved prediction of gambling behavior in a very large dataset^4^. Thus, our model could potentially account for mood fluctuations that occur in everyday life over hours or days. Tracking the neural correlates of these mood fluctuations in the general population raises methodological issues but would be critical to validate our findings that mood level is underpinned by an integration of vmPFC and aIns activity.

Mood level was reflected positively in vmPFC and negatively in aIns, in keeping with the general idea that these two regions are parts of opponent brain systems^23^. As a key component of the reward circuit, later described as the brain valuation system^24,27,28,42,43^, vmPFC was found to signal more positive outcomes with increased activity. By contrast, aIns activity was reported to increase for more negative outcomes^38,44,45^, or for higher costs such as delay, risk or effort^46–50^. This concept of opponency (same variable represented with opposite signs) must be distinguished from domain specificity, which would mean that vmPFC would only respond to positive events, and aIns to negative events^22,23^. Note however that vmPFC and aIns were not mirroring each other: they carried somewhat independent information since they were both explaining some variance in mood level when included in the same regression model. This raises the intriguing question that positive and negative aspects of mood might be independent components, or in other words, that mood could be better described with two dimensions instead of one^51^. From a clinical point of view, it is commonly assumed that the lack of positive affects and the excess of negative affects are largely independent (they are indeed two independent criteria of depression). However, we found no evidence for a simple divide of positive vs. negative mood being represented in vmPFC vs. aIns, since the relationship between mood level and brain activity was roughly linear for both vmPFC and aIns activity, with no apparent change of slope around median mood level. Yet this null finding could come from a limitation of our design, as mood ratings were assigned on a unidimensional scale (from bad mood to good mood), which prevented us to test the presence of two underlying dimensions and to dissociate their neural representations.

Baseline activity in mood-related regions affected choice in a specific manner, with vmPFC increasing the weight of potential gains and aIns increasing the weight of potential losses. These findings bridge two sorts of observations made previously: (1) that enhancing mood promotes risk taking^13^ and (2) that pre-existing activity can affect risky choice^33^. The mechanism we suggest goes beyond a simple additive effect of high baseline activity in risk-promoting regions, since it affects the processing of a specific information (potential gain or loss). We note however that the dissociation is not that clear-cut, as there were trends for vmPFC down-regulating losses and aIns down-regulating gains. This may again mitigate the claim that the vmPFC and aIns are valence-specific regions, and rather supports the notion that vmPFC and aIns are positively and negatively linked to outcome value, respectively.

Importantly, the effect of mood observed and modeled here is a direct impact on choice, and not an indirect impact through biased learning, as in Eldar and Niv’s model^15^. Because mood-inducing and mood-revealing tasks were independent in our paradigm, the impact on choices may be seen as irrational. Yet in an auto-correlated environment, the same effect would be adaptive^16^. For instance, when spring arrives, the appearance of fruits in a given tree predicts the appearance of fruits in other trees. According to our model, getting fruits would enhance mood, by increasing vmPFC and decreasing aIns activity, which would emphasize potential rewards in the calculation of EV, and therefore favor exploratory behavior. This decision can be considered adaptive because exploration would be successful, as fruits are now proliferating in the environment.

Although we believe that we have established interesting links between neural and computational levels, we acknowledge that we took a number of shortcuts. Notably, the transition from feedback to mood was treated at the computational level, so we bypassed the question of how feedback-induced brain responses translate into a sustained change in mood level (or in tonic vmPFC/aIns activity). Similarly, we bypassed the question of how prospect-related brain responses carry over baseline activity so as to modulate the integration of gains and losses and bias the eventual choice. Unfortunately, we are at the limits of what can be done with fMRI, for isolating successive stages of information processing. Further experiments using electrophysiology techniques would be required to further specify the neural mechanisms, in particular for testing whether the neural populations that respond to feedback are the same that distort prospect processing and consequently decision making.

Nevertheless, even such a simple neuro-computational model, where brain activity in key valuation regions is affected by feedback and modulates the relative weighting of gains and losses, might give insight into mood disorders. We can only speculate here, as there may be qualitative differences between normal and pathological mood fluctuations^52^. Our model would predict low vmPFC and high aIns tonic activity in depressed patients, who would overweight potential losses relative to potential gains. This bias would parallel, in value-based decision-making, the negative bias in emotional processing that has been associated with depression^53^. Surprisingly, the behavior of depressed patients in choices involving gains and losses has never been examined, to our knowledge. However, there are hints in the literature that our neural predictions may be correct, even if the neural correlates of mood disorders are still debated^54–60^. Strikingly, vmPFC de-activation has been observed during induced sadness in both remitted depressed and bipolar patients^61,62^. Conversely, aIns hyper-activation, in response to negative stimuli, was observed in depressed patients compared to healthy controls^57,59,63^. We note however that meta-analyses of basal metabolism in depression failed to show reduced levels in the vmPFC, and pointed to even enhanced level in a neighboring subgenual region^58^. Regarding aIns, reduced gray matter volume^64,65^ and low basal metabolism^58,60^ have been associated to depression. Therefore, the question remains open of whether the putative changes in vmPFC and aIns baseline activities could explain the decision to restrict behavior to a core (and safe) minimum, and in particular the reduction of social (and risky) interactions.

## Methods

### Subjects

The study was approved by the Ethics Committee for Biomedical Research (‘Comité de Protection des Personnes’) of the Pitié-Salpêtrière Hospital. Participants were recruited via the Relais d’Information en Sciences Cognitives (RISC) website and screened using an initial telephone interview followed by a personal interview for exclusion criteria: age below 18 or above 39, regular use of drugs or medications, history of psychiatric or neurological disorders, plus (if relevant) contraindications to MRI scanning: pregnancy, claustrophobia, metallic implants. Participants were assessed using personality scales and questionnaires (see Supplementary Methods). All participants gave informed consent prior to partaking in the study. A total of 64 (*n* = 15, 23 and 26 participants in experiment 1, 2 and 3, respectively) healthy, French native-speaker, right-handed volunteers (30 males), aged 18–35 years (mean 23.2 years, s.d. 3.5 years) were recruited. Three subjects were excluded from experiment 3: one because he fell asleep during the experiment and two because of technical problem during acquisition.

### Behavioral tasks

Experiment 1 was composed of 128 trials, divided into eight sessions. Each trial included two tasks: the quiz task and the choice task, separated by a 10–18 s rest period.

Experiment 2 was composed of 256 trials, divided into eight sessions. Each trial included three tasks: the quiz task, the rating task and the choice task. The quiz task and the rating task were separated by a 4–8 s rest period, and the rating task lasted for 5 s.

Experiment 3 was composed of 128 trials, divided into four sessions. Each trial included two tasks: the quiz task and the choice task, separated by a 9–13 s rest period (same duration than between these two tasks in experiment 2).

Quiz task: During the quiz task, a question was displayed on screen for 3.5 s (see example in Fig. 1). The question was randomly selected from a set of 256 possible questions that were adapted from the French version of the “trivial pursuit” game (e.g., In Moby Dick, what is the name of the captain of the Pequod?). Then, four possible answers were presented during up to 4.5 s. Participants were asked to select the correct answer using up and down keys and to confirm their answer using the control key (in the MRI scanner, button responses were used). A 1 s feedback was finally given—either a smiling face with a bell sound or a grimacing face with a buzzer sound. All key/button presses were done with the left hand, as subjects hold the grip in the right hand. In experiment 3 (fMRI), the hand squeezing the grip was counterbalanced across participants.

Unbeknownst to participants, we created episodes of high and low correct response rate. First, questions were sorted by difficulty (assessed by mean accuracy obtained from independent subjects in pilot experiments) and grouped so as to get easy and hard episodes. In experiment 1, each session (32 trials) was assigned to a difficulty level. In experiments 2 and 3, the 7 first and 7 last questions were always of medium difficulty while the 18 middle questions could be either easier or harder. Second, feedback was biased such that a wrong answer could lead to a positive feedback. The proportion of biased feedback (from 0 to 50%) depended on the difficulty of the session (the easier the questions, the more biased the feedback). Note that in a pilot experiment, we ensured with post hoc debriefing that a vast majority of participants remained unaware of this manipulation. The feedback was always positive when the response was correct. The order of difficulty level and proportion bias was fully randomized over sessions for each subject.

Rating task: During this task, which was only included in experiment 2, participants were asked to rate either their confidence or their mood. Note that the difficulty of the upcoming motor challenge was not announced at that time, so the confidence rating was about whether they felt able to perform well whatever the target size. Ratings were triggered by a question appearing on screen (either ‘how do you feel?’ or how do you see your chance of success in the next trial?’. Please note that for the confidence question, participants were explicitly asked to answer about the next motor challenge, together with an analog 21-step scale (10 steps on each side of the center, from low mood/very unconfident to high mood/very confident). Participants had to move the cursor using left and right keys, and to confirm their rating using the control key, within a 5-s delay. They knew that their rating would be maintained on screen until the 5-s delay had elapsed, so they had no reason to hurry up instead of thinking about their estimation. Mood and confidence ratings were both completed in 11 out of 32 trials. In the ten remaining trials, no question was asked but the rest period was increased by 5 s. Therefore, the whole duration from the end of the quiz task to the beginning of the choice task was kept between 9 and 13 s.

Choice task: The choice task began with the presentation of the motor challenge, which lasted for 3–5 s. It was composed of a target window (whose size determined the difficulty, see training section) around 25% of maximal force, and two sets of coins. The upper set (regular coins) represented the potential gain (G, range: 1–5€) in case of success, and the lower set (crossed coins) the potential loss (range: 1–5€) in case of failure (see Fig. 1). Thus, each combination of the three dimensions (target size, gain, and loss) represents a prospect. The sequence of trials was pseudo-randomized such that (1) the three dimensions were orthogonal between them and with the difficulty of quiz questions and (2) all sessions were matched as closely as possible in the mean and variance of these three dimensions.

Then, participants had 1.5 s to make a choice (accept or decline the challenge) using up and down keys (in the MRI scanner, button responses were used). If no choice was made after the 1.5 s delay had elapsed, a penalty of 2€ was inflicted. Just after choice, a picture of a squeezing hand appeared on screen and participants had 2.1 s to initiate their movement. The size of the target (i.e., the difficulty of the trial) was the same whatever the choice. Thus, the choice only determined the amount of money at stake: accepting the challenge meant winning gain prospect or losing loss prospect, whereas declining meant playing for minimal stakes (winning 50 cents or losing 50 cents). For the movement to be as ballistic as possible (like throwing a dart), the participant had to squeeze and release the grip within 400 ms (from the first to the last point above 5% of maximal force). Success or failure depended on whether the peak force was within the target window. No feedback was given to participants about the force or the payoff, to prevent learning effects.

Training: Before the real experiment, participants were familiarized with the handgrip device. Online visual feedback of the force exerted was displayed as a fluid level moving up and down within a thermometer depicted on the screen. The maximal force was recorded for each individual three times in a row (only the highest value was retained). Then three training sessions allowed the participant to learn the required precision movement. These sessions included no quiz or choice. At each trial participants had to produce a movement by squeezing the handgrip, which was followed by a visual feedback showing the force profile over time (as illustrated in Fig. 1). In the first session (10 trials), participants had unlimited time to produce their movement, trying to hit a force target placed at 25% of their maximal force. In the second session (30 trials), the real-time visual feedback was suppressed and participants had to initiate their movement within 2.1 s, as in the real experiment. In the third session (30 trials), participants could win up to 1 euro every trial, depending on the distance of the peak force from the 25% force target. This last training session was used to estimate motor precision for each individual participant and hence to adjust the range of target size. In experiment 1, *S* range was adjusted from [0.6–16] in most precise subjects to [1.6–37.6] in less precise ones (expressed in % of participant maximal force). In experiment 2 and 3, *S* range was somewhat narrower, from [1.5–15] to [3.2–32]. In experiment 3, another precision-training session (similar to the third one) was performed in the scanner to familiarize subjects with the MRI set-up, and to calibrate target sizes for the real experiment. To complete the training, 10 full trials (with the quiz and choice tasks), were finally performed. In order to avoid participants forgetting about the movement, each session of the experiment began with five precision-training trials.

### Behavioral data

Ratings and choices were fitted using computational models. As mood and confidence were sampled every three trials, we linearly interpolated ratings in order to get one data point per trial. In all analyses, mood and confidence ratings were z-scored.

Mood model: We started with a published model^14^ that generates mood level through integration of tasks events, as follows:

$${\mathrm{TML}}\left( t \right) = \omega _0 + \omega _{\mathrm{q1}}\mathop {\sum }\limits_{j = 1}^t \gamma ^{t - j}{{\rm{EV}}_{{\rm{quiz}}}}(t) + \omega _{\mathrm{q2}}\mathop {\sum }\limits_{j = 1}^t \gamma ^{t - j}{{\rm{RPE}}_{{\rm{quiz}}}}(t) \\ \quad+ \omega _p\mathop {\sum }\limits_{j = 1}^t \gamma ^{t - j}{{\rm{EV}}_{{\rm{choice}}}}(t) + \omega _tt$$

where TML is the theoretical mood level, *t* the trial index, whereas *γ* and all *ω* are free parameters (*ω*_0_ is a constant and all other *ω* are weights on the different components; *γ*, with 0 ≤ *γ* ≤ 1, is a forgetting factor that adjusts the influence of recent events relative to older ones). For the quiz task, EV was calculated using the mean accuracy across participants (EV = 2 ∗ accuracy – 1) and RPE was defined as the actual feedback (encoded as 1 when positive and −1 when negative) minus EV. For the choice task, EV was defined as the utility of the chosen option generated by the winning computational model. There was no RPE in this task as no feedback was provided on motor precision.

We explored several variants of this model, according to the following options:

(1) We included versions in which mood was based on the quiz task only, the choice task only, or both tasks. As a sanity check, we also included a null model in which no task had any influence on mood level, which was therefore reduced to a linear function of trial index.

(2) We allowed an asymmetrical influence of positive and negative events on mood, using a free parameter *R* (with *R* > 0) for positive feedback instead of 1.

(3) Following on the suggestion of Eldar and colleagues^15^, we also allowed mood to influence the subjective perception of feedback, as follows:

$$F(t) = {\mathrm{Feedback}}\left( t \right) + \delta \ast {\mathrm{TML}}\left( {t - 1} \right)$$

where *δ* is a free parameter and TML(*t−*1) the TML carried from previous trial, before updating based on the feedback received in the current trial. We assumed that mood effect was additive in order to implement the notion of a ‘rosy outlook’ (a multiplicative effect would imply that a negative feedback is perceived as even worst when one is in a good mood). The modified feedback *F*(*t*) was then used to compute the prediction error:

$${{\rm{RPE}}_{{\rm{quiz}}}}(t) = F(t) - {{\rm{EV}}_{{\rm{quiz}}}}(t)$$

(4) Finally, we tested a simplified version of the model in which only feedback (or its subjective perception *F*, but not EV and RPE taken separately) was taken into account, with a single weight. Formally, this is equivalent to a model in which a single weight *ω*_f_ is used for RPE and EV (*ω*_f_ = *ω*_q1_ = *ω*_q2_), as EV is subtracted from feedback in RPE.

All possible combinations of these options were tested, leading to a total of 18 different models. These models were then compared using Bayesian model selection, in order to identify the best account for rating data.

Choice model: We started with a normative perspective, borrowed from expected utility theory, where potential gains G and losses L are multiplied by probability of success vs. failure. The probability of acceptance was then determined by the difference in expected utility between the two options (accept vs. decline), using a standard softmax function. However, we introduced free parameters to allow some flexibility and potentially capture the effects of mood fluctuations. Notably, we used distinct weights (*k*_g_ and *k*_l_) for the gain and loss components of the utility function:

$${{\rm{Utility}}({\rm{accept}})} = p_{\mathrm{s}} \ast k_{\mathrm{g}} \ast {\mathrm{Gain}} - \left( {1 - p_{\mathrm{s}}} \right) \ast k_{\mathrm{l}} \ast {\mathrm{Loss}}$$

Note that Utility(decline) can be calculated using the same function where gain and loss are replaced by a constant amount (0.5€).

The subjective probability of success *p*_s_ was inferred from the target size. We assumed that participants had a representation of their motor precision following a Gaussian assumption, meaning that the subjective distribution of their peak forces could be defined by its mean—the required 25% of their maximal force—and its width (i.e., standard deviation) captured by a free parameter *σ*. Thus, the probability of success was the integral of this Gaussian bounded by the target window:

$$p_{\mathrm{s}} = \frac{1}{{\sigma \sqrt {2\pi } }}\mathop {\int }\nolimits_{0.25 - \mathrm{Size}/2}^{0.25 + \mathrm{Size}/2} {\mathrm{e}}^{\frac{{ - (x - 0.25)^2}}{{2\sigma ^2}}}{\mathrm{d}}x$$

We also added in the softmax function a constant, in order to capture a possible bias, and a linear function of time on task (trial index *t*), in order to capture fatigue effects:

$$p\left( {\mathrm{accept}},{t} \right) = \frac{1}{{1 + {\mathrm{e}}^{ - ({\mathrm{Utility(accept) - Utility(decline)}} + k_t \ast t + k_0)}}}$$

We tried several variants of this model, with (1) a simplified version in which only Utility(accept) was included in the softmax function, Utility(decline) being considered constant across trials,

(2) a more complex version including curvatures for subjective gain and loss, as in prospect theory^66^. In this last variant, the expected utility of acceptance was, therefore:

$${\mathrm{Utility(accept)}} = p_{\mathrm{s}} \ast k_{\mathrm{g}} \ast {\mathrm{Gain}}^{c_{\mathrm{g}}} - \left( {1 - p_{\mathrm{s}}} \right) \ast k_{\mathrm{l}} \ast {\mathrm{Loss}}^{c_{\mathrm{l}}}$$

where *c*_g_ and *c*_l_ were free parameters (with 0 ≤ *c* ≤ 1).

As the two variants (removing Utility(decline) and introducing curvatures) could be combined, we had four pseudo-normative models. These models were compared to purely descriptive regression models in which expected utility was defined by a linear combination of gain, loss, and target size with all main effects and interactions, the different weights representing free parameters. As each regressor (three main effects, three double interactions, and one triple interaction) could be included or not, we tested 2^7^ = 128 different descriptive models. Acceptance probability was then computed for these descriptive models using the same softmax function as for normative models (without Utility(decline)). Thus, the entire model space included 132 models (128 descriptive and 4 normative).

Model comparison: All models were inverted using a variational Bayes approach under the Laplace approximation^67–69^, implemented in homemade Matlab toolbox (available at http://mbb-team.github.io/VBA-toolbox/). This algorithm not only inverts nonlinear models but also estimates their evidence, which represents a trade-off between accuracy (goodness of fit) and complexity (degrees of freedom)^70^. The log-evidences, estimated for each participant and model, were submitted to a group-level random-effect analysis^69^. This analysis was used to generate exceedance probability, which measures the plausibility that a given model (or model family) is more frequently implemented by participants that any other model (or model family) in the comparison set. The significance of fitted parameters (means of posterior distributions) was tested across participants using one-sample two-tailed *t*-tests or non-parametric test.

### fMRI data

Multiband T2∗-weighted echo planar images (EPIs) were acquired with BOLD contrast on a 3.0 Tesla magnetic resonance scanner. To cover the whole brain with good temporal resolution, we used the following parameters: TR = 1.02 s, 45 slices, 2.5 mm slice thickness, 0.5 mm interslice gap. T1-weighted structural images were also acquired, coregistered with the mean EPI, segmented and normalized to a standard T1 template to allow group level anatomical localization. All data processing and analysis was done using statistical parametric mapping software SPM8 (Wellcome Trust center for NeuroImaging, London, UK) implemented in Matlab. Preprocessing consisted of spatial realignment, normalization using the same transformation as structural images, and spatial smoothing using a Gaussian kernel with a full-width at half-maximum (FWHM) of 8 mm.

Preprocessed individual time series were regressed for each voxel against the following GLMs. The first GLM included five separate categorical regressors for events of the quiz task (at question, answers, and feedback onsets), and those of the choice task (at prospect and question onsets). The prospect-onset regressor was parametrically modulated by its expected utility, as computed in the best computational model of choice. All these regressors were stick function (null duration), except for the last one (question onset), which was a boxcar function. Indeed, as choice and precision movement were separated by 1.5 s only, we model both events by a single boxcar that extended from question onset to the end of precision movement. Another boxcar regressor was included in the model to capture the five precision-training trials that were performed at the beginning of every session. Critically, a last boxcar regressor, encompassing the rest period between quiz and choice tasks (from feedback to prospect onset), was included in the model. It was modulated by the TML, as computed by the best computational model of mood. All regressors were convolved with the canonical hemodynamic response function of SPM8 (without derivative). To correct for motion artifacts, participant-specific realignment parameters were modeled as covariates of no interest. Regression coefficients were estimated at the individual level using the restricted maximum-likelihood estimation. Linear contrasts of regression coefficients were computed at the participant level and then taken to group-level random effect analyses, using one-sample *t*-tests. Statistical maps were family-wise corrected for multiple comparisons at the cluster level.

We ran additional GLM to extract brain activity from specific ROI. ROI were spheres of 8-mm diameter, centered on coordinates independently obtained from a published meta-analysis^42^ on the brain valuation system (MNI coordinates: vmPFC: [2 46 −8]; aIns: [−36 20 −6/40 22 −6]; vS: [−12 12 −6/12 10 −6]; dmPFC: [4 22 44]). The second GLM was similar to the first one except that each rest period (i.e., for each trial) was modeled as a unique, separate regressor. This allowed us to extract brain activity during rest in the different ROI for each trial and each participant. Finally, the third GLM included one event per trial, at the time of prospect onset, convolved with FIR function. This allowed us to extract, for each trial, the BOLD signal at the time of prospect, which is called baseline activity in the results. A last GLM was implemented specifically for Supplementary Fig. 2, in which feedback-onset and prospects-onset regressors were modulated by feedback positivity (positive vs. negative feedback) and expected utility (as computed in the best computational model of choice) respectively. In this last model, the boxcar regressor encompassing the rest period was dropped out. Results were visualized using xjView toolbox (http://www.alivelearn.net/xjview).

### Code availability

All computer codes used during the current study are available from the corresponding author on reasonable request.

### Data availability

The datasets analyzed during the current study are available from the corresponding author on reasonable request.

## Electronic supplementary material

Supplementary Information
