# Supplementary material for: Neuro-computational account of how mood fluctuations arise and affect decision making
Source: Nat Commun. 2018 Apr 26;9:1708. doi: 10.1038/s41467-018-03774-z (PMC5919935; doi:10.1038/s41467-018-03774-z)
Supplement: Supplementary file 1 — Supplementary Information [file 41467_2018_3774_MOESM1_ESM.pdf]

Supplementary Information

Neuro-computational account of how mood fluctuations arise and affect decision making

Vinckier et al.

Supplementary methods: Psychometric assessment

Participants were assessed using personality scales and questionnaires, including the Behavioral Inhibition System / Behavioral Approach System scale (BIS/BAS)[1] and the Big Five Inventory (BFI)[2], as well as Norris visual analog scales (VAS)[3]. VAS was only used in experiment 2 and 3 but twice: first at the very beginning and then at the very end of the whole experimental procedure. In order to reduce the number of possible correlations with computational parameters, we selected 4 sub-scores that may be linked to mood and mood disorders:

- The total scale and the reward responsiveness subscale of the BAS, which had been associated to bipolar disorders and especially to manic symptoms[4,5].
- A depression index that combines neuroticism and conscientiousness (i.e. neuroticism minus conscientiousness sub-scores of the BFI), which are the two personality traits the most strongly associated with depression (in a positive and negative way respectively[6]
- The initial happy/sad VAS score as a proxy for current mood.

However, we did not find any significant association when correcting for multiple correlations, so we did not use personality traits or states to stratify our subjects when reporting the neural and behavioral results.

Supplementary Figure 1. Mood model fit

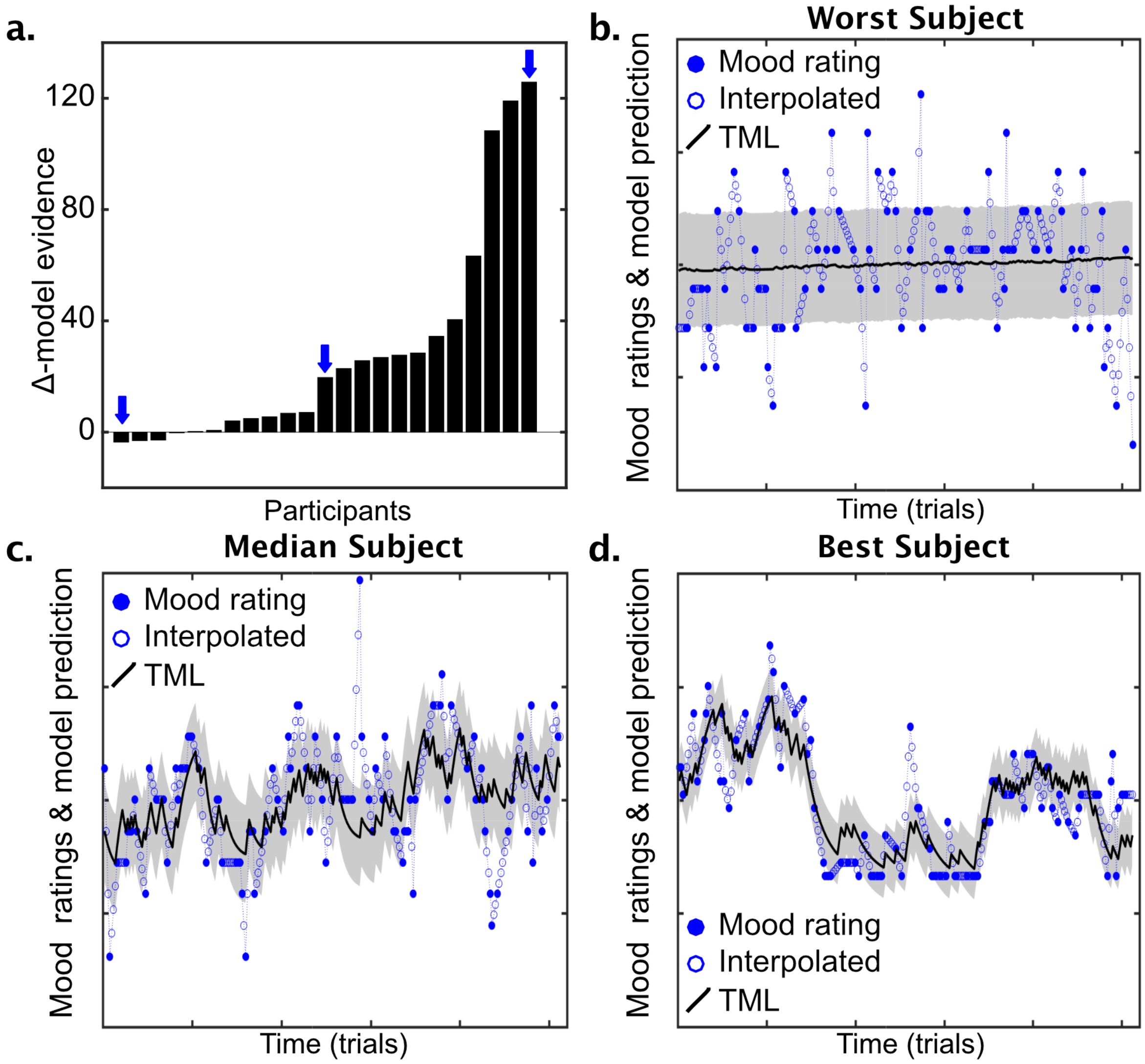

Mood model fit. The different panels show how well the computational model captured fluctuations in mood level. (a) Individual differences in model evidence (variational Bayesian approximation to marginal likelihood) between the best mood model and the control model (in which only time was taken into account). Participants were ranked from left to right in ascending order of model evidence. Blue arrows indicate subjects plotted in the other panels (worst, median and best, from left to right). (b), (c) and (d) Individual examples of mood fluctuations across trials. Blue circles are mood ratings (measured or interpolated) and black lines are theoretical mood level (TML) for subjects showing the worst (b), median (c) and best (d) fit.

Supplementary Figure 2. Feedback- and utility-related brain activations

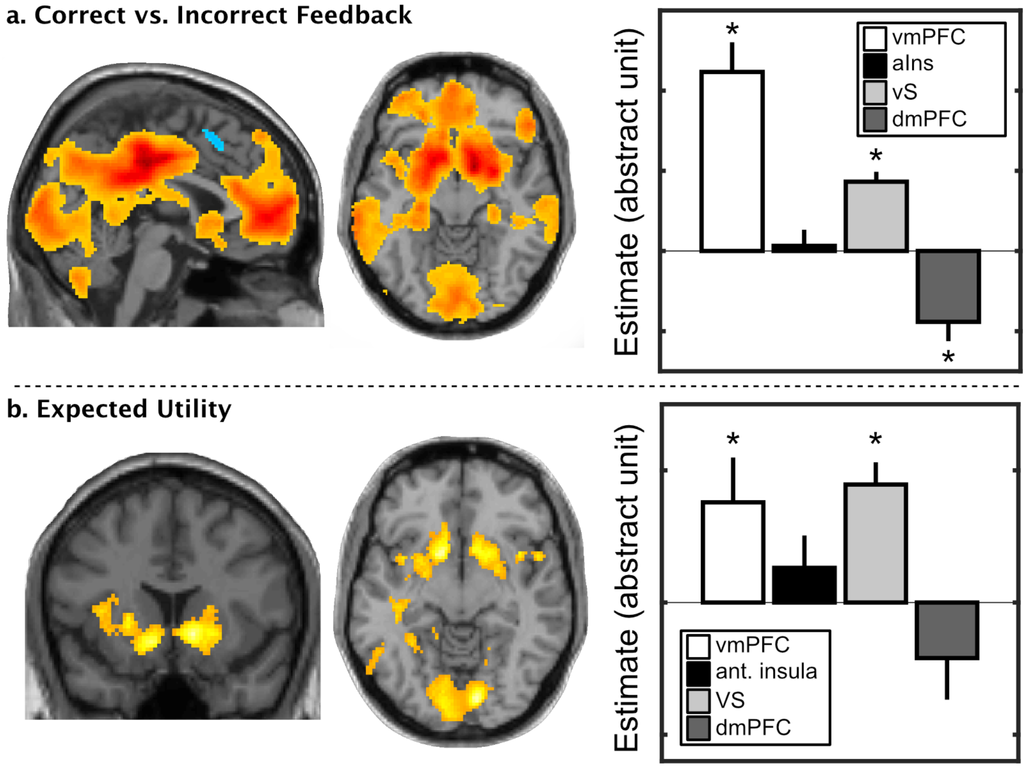

Feedback- and utility-related brain activations a. Statistical parametric map of activations with positive > negative (red) and negative > positive (blue) contrasts, at the time of feedback onset. b. Statistical parametric map of activations with expected utility, as computed by the best choice model (without modulations of k_g and k_l by brain activity). For both maps: cluster generating threshold $p < 0.001$, cluster selection threshold $p < 0.05$ after family-wise error correction for multiple comparisons. Regression coefficients (betas) were extracted in four ROI (vmPFC, anterior insula, ventral striatum and dmPFC). * $p < 0.05$

Supplementary Figure 3. Relationship between baseline activity and theoretical mood level

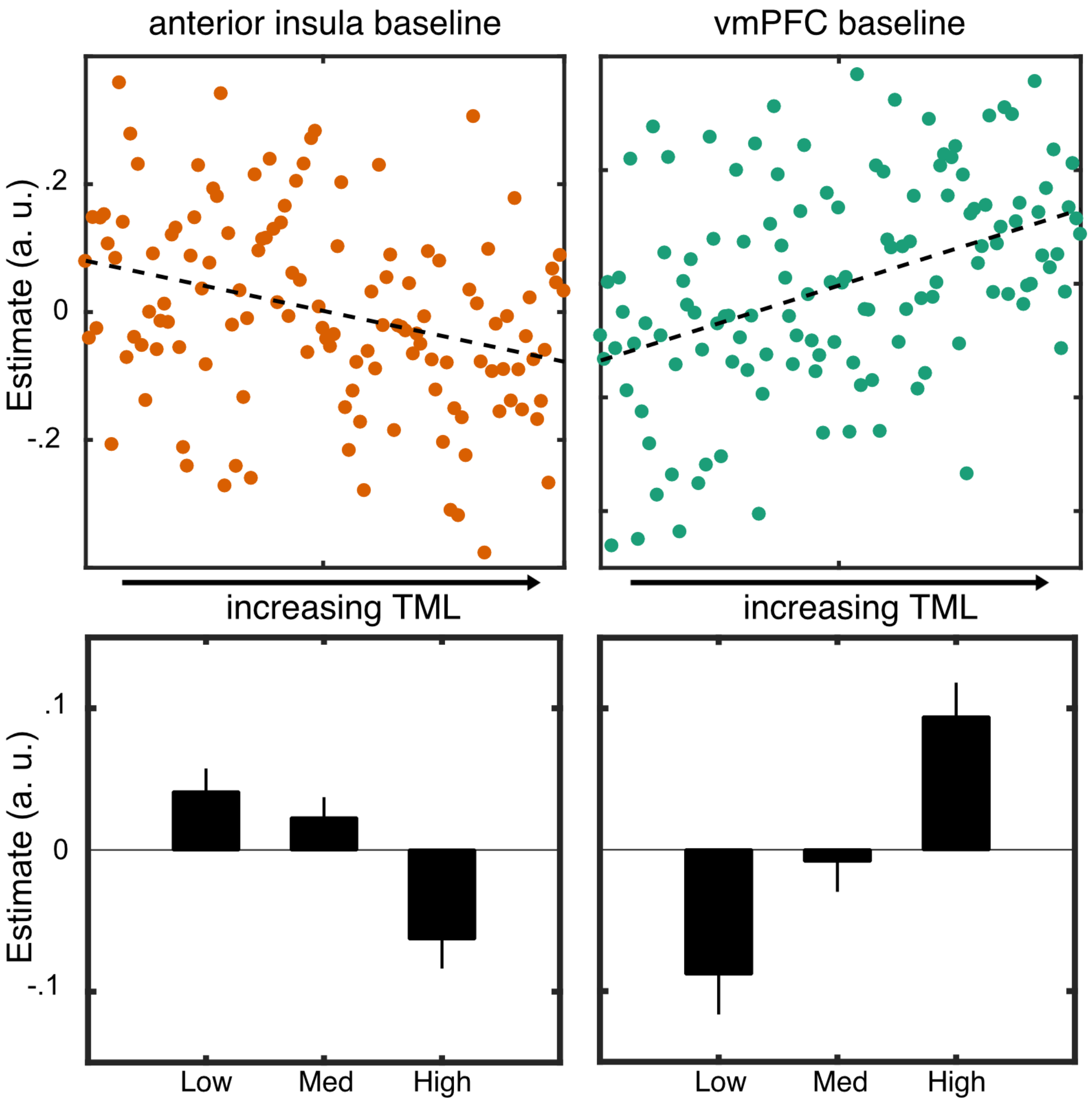

Relationship between baseline activity and theoretical mood level z-scored baseline activity (averaged across all time points preceding prospect onset) in each ROI is shown as a function of TML across all trials (upper graphs) or binned into low/medium/high TML (lower graphs). To specify the relationship, baseline activity was regressed against polynomial expansion of TML. There was no significant association beyond the first-order regressor (all $p > 0.1$), supporting the assumption that the relationship was roughly linear.

Supplementary Table 1.

Brain activations underpinning mood fluctuations					
	Structure	MNI coordinates (x, y, z)			Z-score
Positive	ventromedial prefrontal cortex	4	66	-8	4.96
	medial prefrontal cortex	-16	66	8	4.92
	left inferior parietal lobule	-52	-50	46	5.31
	posterior cingulate cortex	-8	-52	26	5.18
		4	-30	42	3.92
	middle occipital gyrus	-36	-94	0	4.08
Negative	left frontal inferior gyrus	-44	18	30	4.57
	dorsomedial prefrontal cortex	8	24	40	4.29
	left anterior insula *	-34	28	4	3.96
	right anterior insula *	32	24	-4	4.58

Brain activations underpinning mood fluctuations. Related to Fig. 5a Cluster-generating threshold $p < 0.001$, cluster-level threshold $p < 0.05$ family-wise error corrected. * ROI that did not pass cluster-level threshold.

Supplementary References

1 Carver, C. S. & White, T. L. Behavioral inhibition, behavioral activation, and affective responses to impending reward and punishment: The BIS/BAS Scales. *Journal of personality and social psychology* **67**, 319 (1994).

2 Plaisant, O., Courtois, R., Réveillère, C., Mendelsohn, G. & John, O. in *Annales Médico-psychologiques, revue psychiatrique.* 97-106 (Elsevier).

3 Norris, H. The action of sedatives on brain stem oculomotor systems in man. *Neuropharmacology* **10**, 181-191 (1971).

4 Meyer, B., Johnson, S. L. & Winters, R. Responsiveness to threat and incentive in bipolar disorder: Relations of the BIS/BAS scales with symptoms. *Journal of psychopathology and behavioral assessment* **23**, 133-143 (2001).

5 Alloy, L. B. *et al.* High Behavioral Approach System (BAS) sensitivity, reward responsiveness, and goal-striving predict first onset of bipolar spectrum disorders: a prospective behavioral high-risk design. *Journal of abnormal psychology* **121**, 339 (2012).

6 Kotov, R., Gamez, W., Schmidt, F. & Watson, D. (American Psychological Association, 2010).
